# Clinical significance of intratumoral HER2 heterogeneity on trastuzumab efficacy using endoscopic biopsy specimens in patients with advanced HER2 positive gastric cancer

**DOI:** 10.1007/s10120-018-0887-x

**Published:** 2018-10-17

**Authors:** Shusuke Yagi, Takeru Wakatsuki, Noriko Yamamoto, Keisho Chin, Daisuke Takahari, Mariko Ogura, Takashi Ichimura, Izuma Nakayama, Hiroki Osumi, Eiji Shinozaki, Mitsukuni Suenaga, Junko Fujisaki, Yuichi Ishikawa, Kensei Yamaguchi, Ken Namikawa, Yusuke Horiuchi

**Affiliations:** 1 Department of Gastroenterology, The Cancer Institute Hospital of Japanese Foundation for Cancer Research, Tokyo, Japan; 2 Department of Pathology, The Cancer Institute Hospital of Japanese Foundation for Cancer Research, Tokyo, Japan

**Keywords:** HER2 heterogeneity, Trastuzumab, Predictive marker, Endoscopic biopsy specimens, Gastric cancer

## Abstract

**Background:**

We recently reported the clinical significance of intratumoral HER2 heterogeneity on trastuzumab efficacy using surgical specimens; patients with homogeneously HER2 positive gastric cancer benefitted more from trastuzumab. However, the majority of patients are diagnosed by endoscopic biopsy, and surgical specimens are not available in these patients. The aim of this study is to verify clinical significance of HER2 heterogeneity on trastuzumab efficacy using biopsy specimens.

**Methods:**

Eighty-seven patients, who received trastuzumab-based chemotherapy and whose endoscopic biopsy specimens were available for HER2 assessment, were consecutively enrolled. When all tumor cells in all biopsy specimens overexpressed HER2 protein, it was defined as homogeneously HER2 (homo-HER2) positive group, and the others were defined as heterogeneously HER2 (hetero-HER2) positive group. Progression-free survival (PFS), overall survival (OS) and objective response rate (ORR) were evaluated.

**Results:**

Thirty-four patients (39%) were diagnosed as the homo-HER2 group and 53 patients (61%) were the hetero-HER2 group. After the median follow-up period of 17.8 months, the median PFS and OS were 7.6 and 17.8 months, respectively. Significant survival differences were shown between the two groups; the homo-HER2 group showed significantly longer PFS (10.8 vs. 6.1 months, HR 0.469 95% CI 0.29–0.77, *p* = 0.003) and OS (29.3 vs. 14.4 months, HR 0.352 95% CI 0.20–0.61, *p* < 0.001). ORR was 68.6% in this cohort. Higher response rate (85.2% vs 58.1%, *p* = 0.020) and deeper response (− 49.0% vs − 40.0%, *p* = 0.018) were also found in the homo-HER2 group.

**Conclusions:**

Similar to surgical specimens, we verified clinical significance of HER2 heterogeneity on trastuzumab efficacy using endoscopic biopsy specimens.

**Electronic supplementary material:**

The online version of this article (10.1007/s10120-018-0887-x) contains supplementary material, which is available to authorized users.

## Introduction

Gastric cancer is the fifth most common malignancy in the world, and the third common cause of cancer death worldwide [1]. Early detection and early treatment are being conducted mainly in East Asia; however, the prognosis of patients with advanced gastric cancer is still insufficient [2–6]. Therefore, development of new molecular agents and treatment strategy are needed for overcoming difficult situations.

Human epidermal growth factor receptor 2 (HER2) is involved in tumor cell proliferation, migration and differentiation [7]. It has been reported that HER2 amplification or overexpression is found in 7–34% of gastric cancer [7–10]. The ToGA trial demonstrated superiority of additional trastuzumab to the standard chemotherapy as first line in patients with HER2 positive gastric cancer [11]. Based on this result, trastuzumab-based chemotherapy has become a standard care in first-line treatment for HER2 positive advanced gastric cancer. Previous clinical trials have shown that the median progression-free survival (PFS) was around 6 months across trials and various response rates were from 47 to 82.1% [11–14]. These data imply that there is a substantial proportion who do not respond to trastuzumab and, once responded, the majority of patients relapse within several months. Therefore, it is needed to identify the appropriate patients who benefit more from trastuzumab and mechanism of resistance.

Several possible biomarkers, including HER2 gene amplification levels, serum HER2 protein levels, serum neuregulin1 levels and HER2 protein levels by proteomic analysis, have been proposed as a predictive marker for trastuzumab efficacy [13, 15–18]. However, these methods are expensive and require additional analysis. As a result, these markers are not widely accepted in clinical practice. Intratumoral HER2 heterogeneity is frequently observed with in gastric cancer [19–23]. We recently reported clinical significance of HER2 heterogeneity on trastuzumab efficacy using surgical specimens; patients whose tumor homogeneously HER2 overexpressed benefited more from trastuzumab compared with heterogeneously HER2 overexpressed [24]. However, the majority of patients who receive trastuzumab-based chemotherapy are unresectable and are only available for biopsy specimens. Clinical significance of HER2 heterogeneity using biopsy specimens is unclear. The aim of this study is to verify the clinical significance of HER2 heterogeneity on trastuzumab efficacy using biopsy specimens.

## Materials and methods

### Study cohort

Patients who received trastuzumab-based chemotherapy as first-line treatment in The Cancer Institute Hospital of Japanese Foundation for Cancer Research from March 2011 to March 2016 were subjected. Among them, patients whose endoscopic biopsy specimens taken from primary tumor were available for HER2 assessment were consecutively enrolled. This study was approved by the Institutional Review Boards (No. 2015-1029) and all patients signed an informed consent for the analysis of molecular correlates.

### Treatment schedule

Trastuzumab was given by intravenous infusion at a dose of 8 mg/kg on day 1 of the first cycle, followed by 6 mg/kg every 3 weeks. Capecitabin 1000 mg/m^2^ was given orally twice a day for 14 days followed by a 1-week rest. S-1 was given orally twice a day for 14 days followed by a 1-week rest at a dose based on body surface area (< 1.25 m^2^, 40 mg; ≥ 1.25 to < 1.5 m^2^, 50 mg; ≥ 1.5 m^2^, 60 mg). Fluorouracil 800 mg/m^2^ per day was given by continuous intravenous infusion on day 1 of each cycle. Cisplatin 80 mg/m^2^ on day 1 was given by intravenous infusion, or oxaliplatin was administrated as a 100–130 mg/m^2^ infusion and they were given every 3 weeks. Treatment was repeated until disease progression, development of unacceptable toxicity, or patient withdrawal of consent. Computed tomography assessments were repeated every 6–8 weeks and RECIST ver1.1 was used to define all responses [25]. Clinical information was retrieved from electronic medical charts.

### Immunohistochemistry

HER2 assessment was performed on whole sections of formalin-fixed paraffin-embedded tumor blocks of endoscopic biopsy specimens. Immunohistochemistry (IHC) was conducted using an automatic immunostainer (BenchMark ULTRA^®^ Tucson, AZ, USA) and the primary antibody used was anti-HER2 (4B5) (10,798; rabbit monoclonal; Ventana, Tucson, AZ, USA) according to the manufacturer’s instructions. HER2 IHC was scored using a four-grade scale (0/1+/2+/3+) according to scoring scheme [8] as follows: 0, no reactivity or membranous reactivity in any cells; 1+, faint or barely perceptible membranous reactivity in at least 1 cluster of ≥ 5 tumor cells; 2+, weak to moderate complete, basolateral or lateral membranous reactivity in at least 1 cluster of ≥ 5 tumor cells; and 3+, strong complete, basolateral or lateral membranous reactivity in at least 1 cluster of ≥ 5 tumor cells. IHC score of 3+ or IHC score of 2+ with FISH positivity was defined as HER2 positive, whereas IHC score of 0 or 1+, or IHC score of 2+ with FISH negativity was defined as HER2 negative.

### Evaluation of intratumoral HER2 heterogeneity

**Fig. 1:**
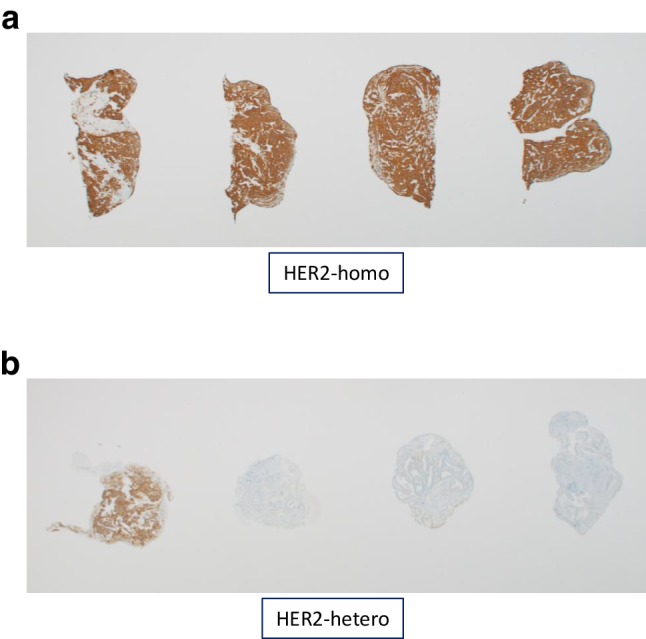
Representative images of homogeneously HER2 positive gastric cancer. **a** Hematoxylin-eosin stains shows differentiated adenocarcinoma. HER2 IHC shows that almost all tumor cells overexpress HER2 protein in each specimen corresponding to HE stains. Representative images of heterogeneously HER2 positive gastric cancer. **b** HER2 IHC shows that HER2 protein overexpressed in some specimens

In patients with HER2 positive gastric cancer diagnosed by HER2 scoring system, we evaluated intratumoral HER2 heterogeneity in all biopsy specimens containing tumor cells. Because there is no guideline for the assessment of intratumoral HER2 heterogeneity in gastric cancer, we defined intratumoral HER2 heterogeneity as follows: when all tumor cells in all biopsy specimens stained HER2 IHC3+ or IHC2+, it was defined as homogeneously HER2 (homo-HER2) positive group, and the others were defined as heterogeneously HER2 (hetero-HER2) positive group (Fig. 1). A single pathologist (NY) reviewed all slides without any clinical information.

### Statistical analysis

Progression-free survival (PFS), overall survival (OS) and objective response rates (ORRs) were evaluated. PFS was defined between the date of initial chemotherapy and first documented progression or death from any cause. OS was defined between the date of initial chemotherapy and death from any cause. If patients did not meet any endpoints until 31 March 2018, they were censored at the time of last contact. When patients underwent conversion surgery, they were censored at the time of the date of surgery in PFS. Survival curves for PFS and OS were estimated using the Kaplan–Meier method. Prognostic value of intratumoral HER2 heterogeneity using endoscopic specimens and other clinical factors were assessed using univariant and multivariate analysis by Cox proportional hazard model. Patient characteristics and response rates were evaluated by Fisher’s exact test. Depth of response was compared using Mann–Whitney *U* test. The level of significance was set to *p* < 0.05 and all statistical tests were two-sided. All statistical analyses were performed with EZR version 1.36 (Saitama Medical Center, Jichi Medical University, Saitama, Japan), which is a graphical user interface for R (The R Foundation for Statistical Computing, Vienna, Austria) [26].

## Results

### Patient characteristics

One-hundred and thirty-three patients received trastuzumab-based chemotherapy as first-line treatment in our institute from March 2011 to March 2016. Among them, 87 patients whose biopsy specimens were available for HER2 assessment were enrolled. (EMS1) The median number of biopsy specimens was 4. The median number of fragments containing cancer cells was 4 (range 1–8), and the median number of HER2 positive fragments (HER2 2+ or 3+) was 3. The median number of HER2 3+ fragment was 2 (ESM 2).

The clinicopathologic characteristics of 87 patients are shown in ESM 3. Briefly, 78 patients diagnosed with HER2 IHC 3+ (89.7%). 33 patients (39.1%) were diagnosed as homo-HER2 positive and 53 patients (60.9%) were diagnosed as hetero-HER2 positive. Therefore, the rate of HER2 heterogeneity in this cohort was 60.9%. Although most patients (92.0%) were treated with platinum doublet plus trastuzumab regimen, seven patients (8%) received without platinum because of old age. Second-line therapy was performed on 65 patients (74.7%). Nine patients (10.3%) underwent conversion surgery and seven of them were received adjuvant S-1 after conversion surgery. In three of them, trastuzumab was used as adjuvant chemotherapy combined with S-1.

Table 1 shows a comparison of demographics between the homo-HER2 and the hetero-HER2 positive groups. There was no significant difference across most clinical factors between the two groups. However, the homo-HER2 positive group was younger and received more prevalently platinum-based chemotherapy than the hetero-HER2 positive group. The induction rates of second-line treatment were comparable between the two groups. In second-line chemotherapy, monoclonal antibodies that target HER2 were administered in 14 patients. Six patients were in the homo-HER2 positive group and eight patients in the hetero-HER2 positive group, respectively. Two patients in the homo-HER2 positive group received T-DM1 as a clinical trial, and the other 12 patients were given trastuzumab combined with paclitaxel.

**Table 1:** Comparison of patients’ characteristics between homo-HER2 and hetero-HER2 positive groups in this study

Characteristics	Hetero-HER2 *n* = 53 (%)	Homo-HER2 *n* = 34 (%)	*p* value
Age	68	62.5	0.029
≥ 67	32 (60.4)	12 (35.3)	
< 66	21 (39.6)	22 (64.7)	
Sex			0.357
Male	37 (69.8)	20 (58.8)	
Female	16 (30.2)	14 (41.2)	
ECOG PS			0.481
0	34(64.2)	25 (73.5)	
1	19 (35.8)	9 (26.5)	
Primary tumor site			0.335
EGJ	13 (24.5)	12 (35.3)	
Stomach	40 (75.5)	22 (64.7)	
Histological type			0.362
Differentiated type	31 (58.5)	24 (70.6)	
Undifferentiated type	22 (41.5)	10 (29.4)	
Visceral metastasis			1.000
Yes	28 (52.8)	18 (52.9)	
No	25 (47.2)	16 (47.1)	
Previous gastrectomy			1.000
Yes	17 (32.1)	11 (32.4)	
No	36 (67.9)	23 (67.6)	
Platinum-based			0.039
Yes	46 (86.8)	34 (100)	
No	7 (13.2)	0 (0.0)	
Conversion surgery			0.304
Yes	4 (7.5)	5 (14.7)	
No	49 (92.5)	29 (85.3)	
Second line chemotherapy			1.000
Yes	40 (75.5)	25 (73.5)	
No	13 (24.5)	9 (26.5)	
Beyond HER2 targeted therapy			0.772
Yes	8 (15.1)	6 (17.6)	
No	45 (84.9)	28 (82.4)	
HER2 status			0.011
IHC 3+	44 (83.0)	34 (100)	
IHC 2+/FISH positive	9 (17.0)	0 (0.0)	
CEA (ng/ml)			0.498
≥ 5.0	31 (58.5)	23 (67.6)	
< 5.0	22 (41.5)	11 (32.4)	
CA 19-9 (U/ml)			0.657
≥ 37.0	29 (54.7)	21 (61.8)	
< 37.0	24 (45.3)	13 (38.2)	

*Hetero-HER2* Heterogeneously HER2 positive, *Homo-HER2* homogeneously HER2 positive, *EGJ* esophagogastric junction, *ECOG PS* Eastern Cooperative Oncology Group Performance Status, *FISH* fluorescence in-situ hybridisation, *IHC* immunohistochemistry

### Survival

After the median follow-up period of 17.8 months, the median PFS and OS were 7.6 months [95% CI 5.9–9.5] and 17.8 months [95% CI 14.4–21.7], respectively. The median number of cycles of trastuzumab therapy was 9 (range 1–71) in this cohort.

**Fig. 2:**
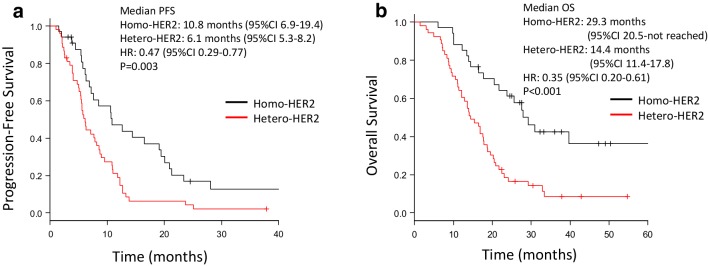
Progression-free survival and overall survival. Significantly longer progression-free survival is seen in the homo-HER2 positive group compared with the hetero-HER2 positive group (**a**). Significantly longer overall survival is also seen in the homo-HER2 positive group compared with the hetero-HER2 positive group (**b**)

Patients with the homo-HER2 positive group showed better survivals; the median PFS was 10.8 months [95% CI 6.9–19.4] in the homo-HER2 positive group compared with 6.1 months [95% CI 5.3–8.2] in the hetero-HER2 positive group (HR 0.47 95% CI 0.29–0.77; *p* = 0.003) (Fig. 2a). The median OS was 29.3 months [95% CI 20.5–not reached] in the homo-HER2 positive group compared with 14.4 months [95% CI 11.4–17.8] in hetero-HER2 (HR 0.35 95% CI 0.20–0.61; *p* < 0.001) (Fig. 2b).

Univariate analysis was performed with respect to clinicopathological factors, including age, sex, performance status, primary tumor site, histopathologic type, HER2 status, number of metastatic site, visceral metastasis, platinum-based chemotherapy, the level of CEA and CA19-9. Among them, platina-based chemotherapy was only associated with PFS and OS (ESM 4). Multivariate analysis stratified by age and platinum-based chemotherapy revealed that HER2 heterogeneity retained to be significant (Table 2).

**Table 2:** Survival outcomes by multivariate analyisis

Covariates	PFS		OS	
	HR 95% (CI)	*p* value	HR 95% (CI)	*p* value
Age		0.046		0.22
67 ≥ (44)	1 (reference)		1 (reference)	
< 66 (43)	1.69 (1.01–2.84)		1.39 (0.82–2.35)	
Platinum-based		0.002		0.01
Yes (80)	1 (reference)		1 (reference)	
No (7)	5.63 (2.26–13.99)		3.15 (1.32–7.55)	
HER2 heterogeneity		< 0.001		< 0.001
Yes (53)	1 (reference)		1 (reference)	
No (34)	0.43 (0.25–0.74)		0.36 (0.20–0.63)	

*HR* Hazard ratio, *95% CI* 95% confidential interval, *PFS* progression-free survival, *OS* overall survival

### Response

**Fig. 3:**
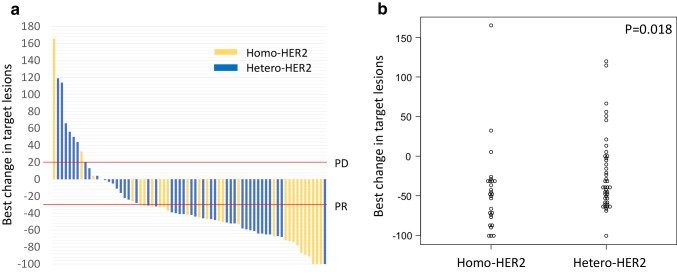
Best change from baseline in size of target lesion. Water-fall plot reveals that patients in the homo-HER2 positive group obtain deeper tumor shrinkage compared with the hetero-HER2 positive group (**a**). Scatter plot shows statistically significant difference in tumor shrinkage between two groups (*p* = 0.018) (**b**)

Tumor response was evaluated in 70 patients; 27 patients were in the homo-HER2 positive group and 43 patients were in the hetero-HER2 positive group. Overall responses were complete response in 5 patients (7.1%), partial response in 43 (61.4%), stable disease in 13 (18.6%), and progression disease in 9 (12.9%), respectively. Therefore, ORR and disease control rate in this cohort were 68.6% and 87.1%, respectively. Higher response rate was shown in the homo-HER2 positive group than in the hetero-HER2 positive group (85.2% [95% CI 66.3–95.8] vs 58.1% [95% CI 42.1–73]; OR 4.06 [95% CI 1.110–18.961]; *p* = 0.020; Table 3). In addition, the median depth of response was also significantly deeper in the homo-HER2 positive group than in the hetero-HER2 positive group (− 49.0% [IQR − 76.0 to − 32.5] vs − 40.0% [IQR − 55.0 to − 2.0]; *p* = 0.018; Fig. 3).

**Table 3:** Best overall response

	Hetero-HER2	Homo-HER2	*p* value
	*n* = 43 (%)	*n* = 27 (%)	
CR	1 (2.3)	4 (14.8)	
PR	24 (55.8)	19 (70.4)	
SD	11 (25.6)	2 (7.4)	
PD	7 (16.3)	2 (7.4)	
ORR	25/43 (58.1)	23/27 (85.2)	0.02

*CR* Complete response, *PR* partial response, *SD* stable disease, *PD* progression disease, *ORR* objective response rate, *Hetero-HER2* heterogeneously HER2 positive, *Homo-HER2* homogeneously HER2 positive

### Association between the number of biopsy and survivals

To seek the optimal number of biopsy specimens, especially for the homo-HER2 positive group assessment, we examined a correlation between the number of biopsy specimens containing tumor cell and survival in each group. ESM 5 are scatter plots showing the associations between the number of biopsy specimens and the median PFS and OS in each group. There was no correlation between the number of biopsy specimens and survival in all groups. It seems that less diverse PFS and OS are shown in the hetero-HER2 group, particularly, PFS, whereas diverse PFS and OS are shown in the homo-HER2 positive group.

## Discussion

Intratumoral HER2 heterogeneity is frequently seen in gastric cancer; however, its clinical significance on trastuzumab efficacy was unclear. We recently reported clinical significance of HER2 heterogeneity on trastuzumab efficacy using surgical specimens [24]. However, clinical significance of HER2 heterogeneity in endoscopic biopsy specimens was still unclear. In the present study, we verified clinical significance of HER2 heterogeneity using biopsy specimens; in the same way to surgical specimens, patients with homogeneously HER2 positive gastric cancer showed longer survival, higher response rate and a deeper response than those with heterogeneous HER2 positive gastric cancer. Our data suggest that HER2 heterogeneity is a useful biomarker for predicting trastuzumab efficacy regardless of the types of tumor sample.

The incidence of HER2 heterogeneity evaluated by IHC is widely reported from 39.0 to 75.4% [19–23]. Various types of sample including surgical specimens, biopsy specimens and tissue-microarray were used in previous studies. In addition, un-unified cut-off values from 30 to 100% have been adopted for HER2 heterogeneity assessment. No guidelines for HER2 heterogeneity assessment and different types of sample possibly cause widely different reported incidences of HER2 heterogeneity. In this study, we used endoscopic biopsy specimens and set the cut-off value as 100%: The homo-HER2 positive group was defined when all tumor cells in all biopsy specimens contained tumor cell over-expressed HER2 protein. When using this cut-off value, the incidence of HER2 heterogeneity was 60.9% in this cohort. This incidence is supported by Korean data by Ahn et al.; the incidence was 61.5% when used biopsy specimens and the same cut-off value [27]. Similar incidence between our data and Korean data under the same condition suggests that this cut-off value for HER2 heterogeneity assessment is appropriate and should be used in future studies.

We showed superior survival in the homo-HER2 positive group using biopsy specimens; however, survival in the homo-HER2 positive group in this study seems to be shorter than those in a previous report using surgical specimens [24]. The comparison of survivals between surgical specimens and biopsy specimens is shown in Table 4. Despite having the same HER2 expression pattern, why are survivals in biopsy specimens shorter than those in surgical specimens? Ahn et al. carefully evaluated the clinical impact of HER2 heterogeneity on HER2 status assessment using surgical specimens and paired biopsy specimens obtained from more than 700 patients [27]. According to this report, 34.3% of the heterogeneous HER2 positive cases in surgical specimens were homogeneous HER2 positive cases in biopsy specimens, suggesting lower specificity and positive predictivity of biopsy specimens for the homo-HER2 positive group assessment. Therefore, some patients of the homo-HER2 positive group in this study may have been discrepancies. On the other hand, this report also described that HER2 heterogeneity found in biopsy specimens was significantly correlated with its surgical specimens [27]. This data support comparable survivals between this study and a previous report in the hetero-HER2 positive group.

**Table 4:** Comparison survivals between surgical specimens and biopsy specimens

	Biopsy specimens	Surgical specimens
mPFS		
Homo-HER2	10.8 (95% CI 6.9–19.4)	20.0 (95% CI 17.8–22.2)
Hetero-HER2	6.1 (95% CI 5.3–8.2)	6.0 (95% CI 2.3–9.7)
mOS		
Homo-HER2	29.3 (95% CI 20.5–NR)	NR
Hetero-HER2	14.4 (95% CI 11.4–17.8)	14.0 (95% CI 11.9–16.1)

*mPFS* Median progression-free survival, *mOS* median overall survival, *NR* not reached, *Homo-HER2* homogeneously HER2 positive, *Hetero-HER2* heterogeneously HER2 positive

We expected that there would be a positive correlation between the number of biopsies and survival in the homo-HER2 positive group; however, no correlation was found. These data suggest that, even if we increase the number of biopsies, specificity and positive predictivity of biopsy specimens of assessment for the homo-HER2 positive group may not increase. Biopsy specimens would have limitation of diagnostic ability to obtain whole tumor biologic property in gastric cancer. As a result, it is difficult to select appropriate patients in clinical trials. This sampling limitation and molecular heterogeneity in gastric cancer may be attributed to the negative results of clinical trials for anti-HER2 agents such as lapatinib, T-DM1 and pertuzumab [28–30]. On the other hand, less diverse survivals were shown in the hetero-HER2 positive group, particular in PFS. Namely, once diagnosed as the hetero-HER2 positive gastric cancer, these patients may expect only modest treatment effect. Taken together, it seems that HER2 heterogeneity in surgical specimens is a useful biomarker as a positive predictor for anti-HER2 agents, while HER2 heterogeneity in biopsy specimens should be used as a negative predictor.

There are several limitations in this study. First of all, this is a retrospective study from a single institution. Secondly, the optimal cut-off value and ideal number of biopsy specimens for HER2 heterogeneity assessment are still not concluded. Finally, the mechanism of resistance to trastuzumab in heterogeneous HER2 positive gastric cancer is unclear. Nevertheless, different from biomarkers previously reported, HER2 heterogeneity is a simple and inexpensive biomarker. We recommend that a pathologist should consider evaluating intratumoral HER2 heterogeneity when assessing HER2 status.

In conclusion, we verified that intratumoral HER2 heterogeneity using biopsy specimens showed clinical significance on trastuzumab efficacy. Prospective clinical trials and further molecular correlate analysis are warranted.

## Electronic supplementary material

Below is the link to the electronic supplementary material.

Supplementary material 1 (PPTX 42 KB)

Supplementary material 2 (PPTX 45 KB)

Supplementary material 3 (XLSX 12 KB)

Supplementary material 4 (XLSX 15 KB)

Supplementary material 5 (PPTX 86 KB)
