# Supplementary material for: Clinical significance of intratumoral HER2 heterogeneity on trastuzumab efficacy using endoscopic biopsy specimens in patients with advanced HER2 positive gastric cancer
Source: Gastric Cancer. 2018 Oct 17;22(3):518–25. doi: 10.1007/s10120-018-0887-x (PMC6476840; doi:10.1007/s10120-018-0887-x)
Supplement: Supplementary file 1 — Supplementary material 1 (PPTX 42 KB) [file 10120_2018_887_MOESM1_ESM.pptx]

## Slide 1
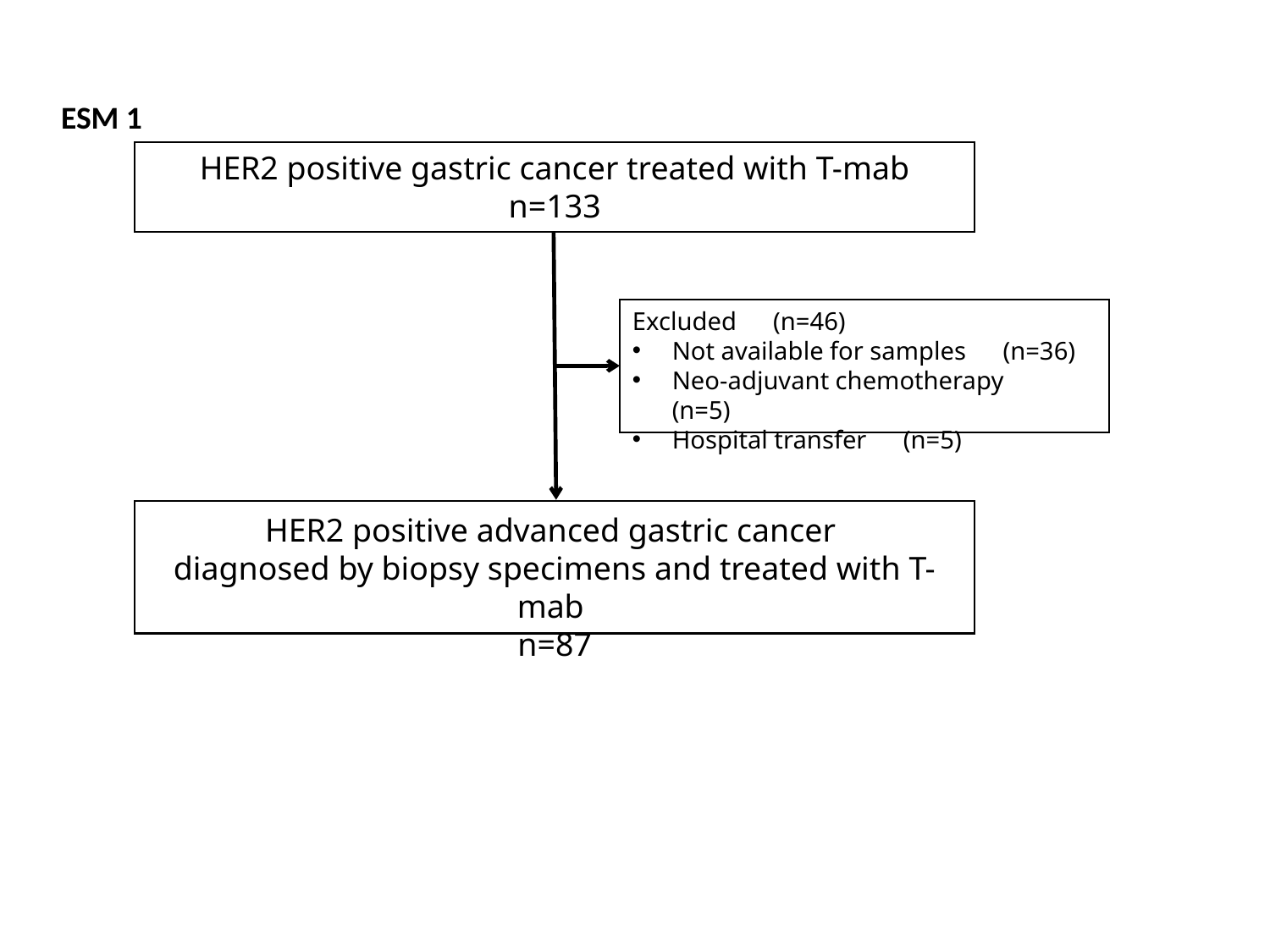

ESM 1
HER2 positive gastric cancer treated with T-mab
n=133
Excluded　(n=46)
Not available for samples　(n=36)
Neo-adjuvant chemotherapy　(n=5)
Hospital transfer　(n=5)
HER2 positive advanced gastric cancer
diagnosed by biopsy specimens and treated with T-mab
n=87
