# Supplementary material for: Clinical significance of intratumoral HER2 heterogeneity on trastuzumab efficacy using endoscopic biopsy specimens in patients with advanced HER2 positive gastric cancer
Source: Gastric Cancer. 2018 Oct 17;22(3):518–25. doi: 10.1007/s10120-018-0887-x (PMC6476840; doi:10.1007/s10120-018-0887-x)
Supplement: Supplementary file 2 — Supplementary material 2 (PPTX 45 KB) [file 10120_2018_887_MOESM2_ESM.pptx]

## Slide 1
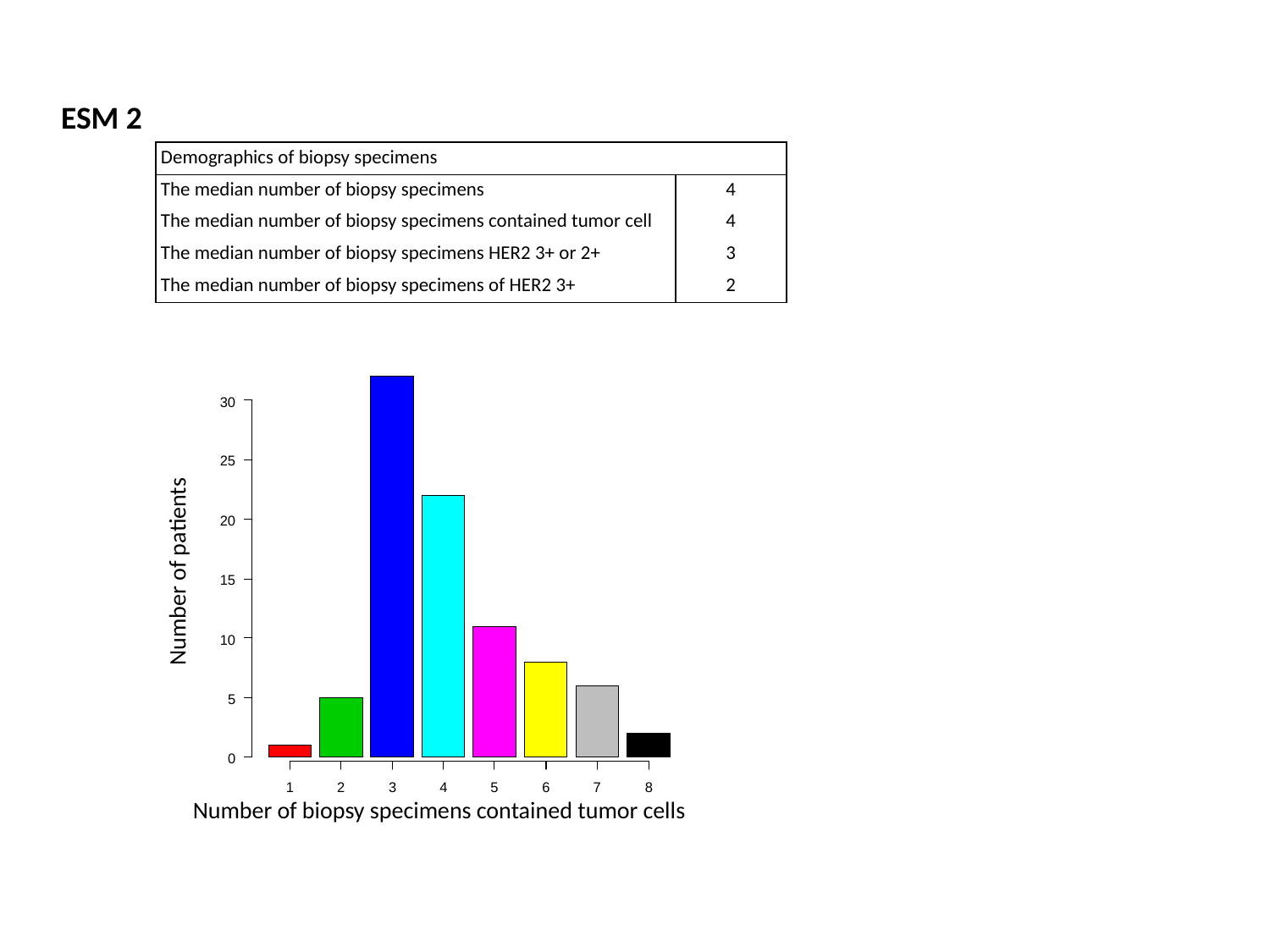

ESM 2
| Demographics of biopsy specimens | |
| --- | --- |
| The median number of biopsy specimens | 4 |
| The median number of biopsy specimens contained tumor cell | 4 |
| The median number of biopsy specimens HER2 3+ or 2+ | 3 |
| The median number of biopsy specimens of HER2 3+ | 2 |
Number of patients
Number of biopsy specimens contained tumor cells
