# Supplementary material for: Clinical significance of intratumoral HER2 heterogeneity on trastuzumab efficacy using endoscopic biopsy specimens in patients with advanced HER2 positive gastric cancer
Source: Gastric Cancer. 2018 Oct 17;22(3):518–25. doi: 10.1007/s10120-018-0887-x (PMC6476840; doi:10.1007/s10120-018-0887-x)
Supplement: Supplementary file 5 — Supplementary material 5 (PPTX 86 KB) [file 10120_2018_887_MOESM5_ESM.pptx]

## Slide 1
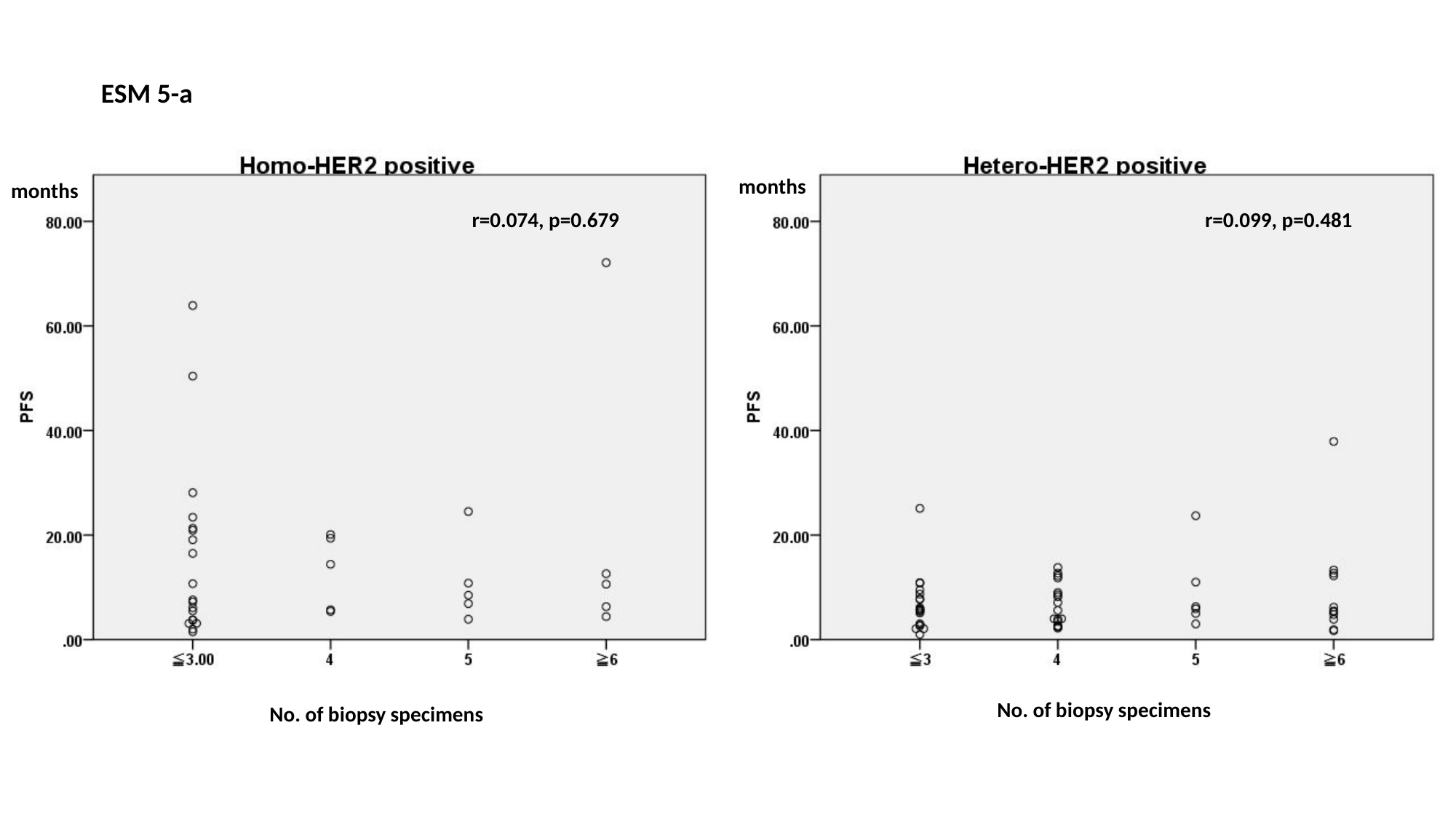

ESM 5-a
months
months
r=0.074, p=0.679
r=0.099, p=0.481
No. of biopsy specimens
No. of biopsy specimens

## Slide 2
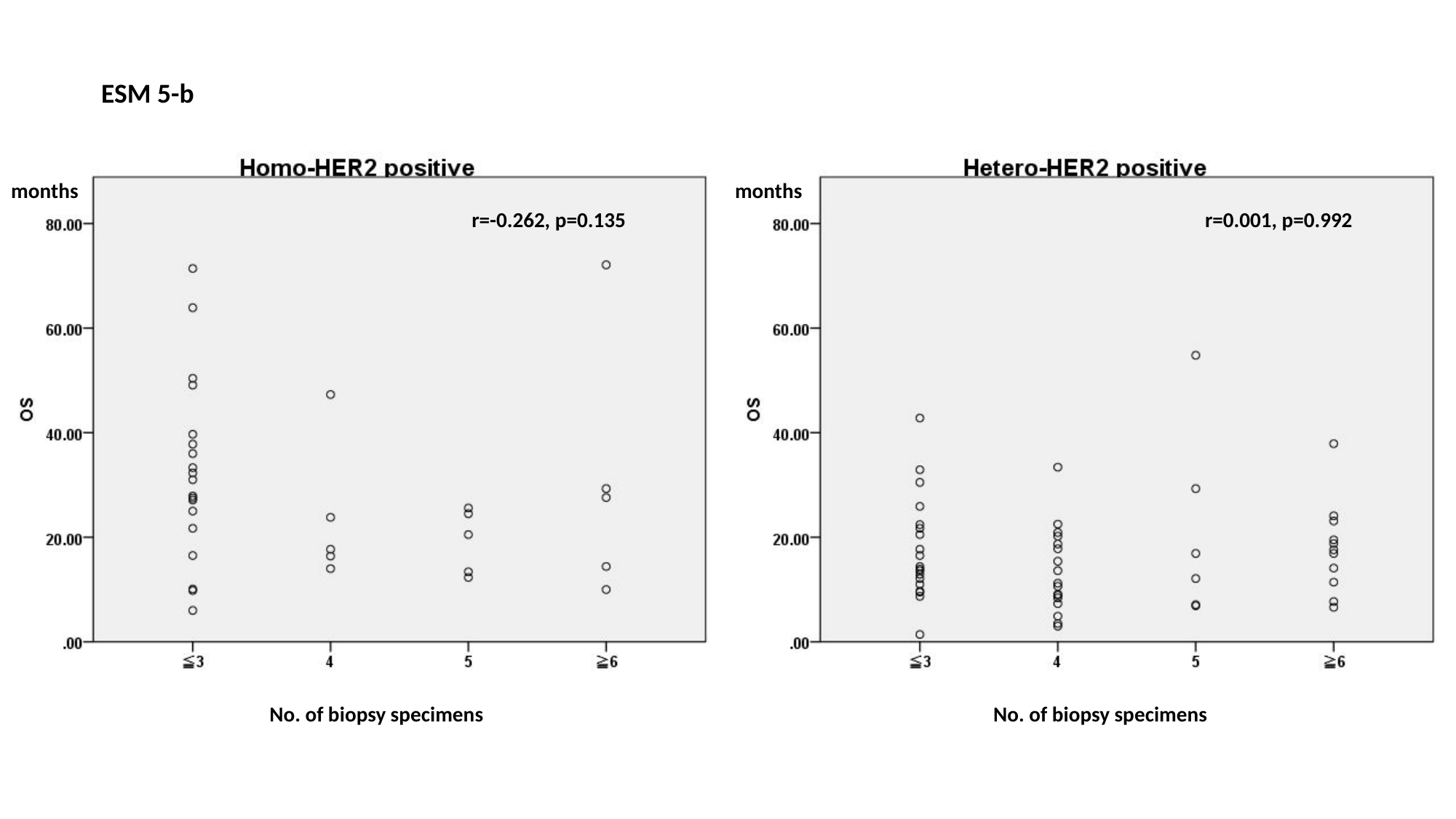

ESM 5-b
months
months
r=-0.262, p=0.135
r=0.001, p=0.992
No. of biopsy specimens
No. of biopsy specimens
